# Compassion and burnout syndrome in medical students from the Colombian Caribbean coast

**DOI:** 10.1192/j.eurpsy.2023.771

**Published:** 2023-07-19

**Authors:** E. P. Ruiz Gonzalez, A. Krikorian, A. M. Romero, M. Lemos, J. P. Roman

**Affiliations:** 1 Universidad Pontifica Bolivariana, Montería; 2 Universidad Pontifica Bolivariana, Medellin; 3 Universidad de Córdoba, Montería; 4 Universidad EAFIT, Medellin, Colombia

## Abstract

**Introduction:**

Compassion is expected to be a characteristic present in medical students, since it is a key element in subsequent professional practice (Blanco et al., 2021). However, during the degree, students go through various demands that can generate burnout syndrome (Amor et al., 2020) and as a consequence a decrease in compassion. In this sense, it is important to provide empirical evidence on the possible relationship between these two constructs, in order to generate support that allows the implementation of mental health promotion strategies.

**Objectives:**

Analyze the relationship between compassion and burnout syndrome in medical students.

**Methods:**

This study was done through a cross-sectional study of correlational scope in 250 medical students. The Compassion Scales developed by Gilbert (Gilbert et al., 2017) were used; they assess three general factors (Self-compassion, Compassion for others, and Compassion from others), However, in this study, we used only the self-compassion and compassion for others scales.

**Results:**

The results revealed a statistically significant, positive correlation between personal accomplishment and self-compassion. In the case of emotional exhaustion and depersonalization, significant associations were also found with self-compassion and compassion for others, but of a negative magnitude (Table 1).

**Image:**

**Figure:**
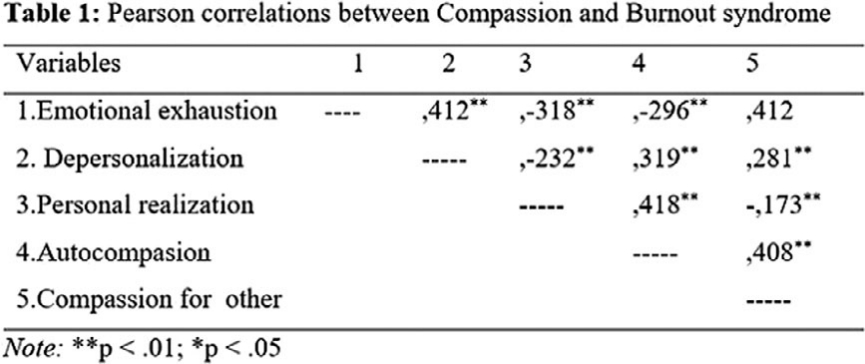

**Conclusions:**

It was concluded that the higher the levels of self-compassion of the medical students evaluated, the greater their personal fulfillment. On the other hand, the lower the levels of self-compassion and compassion for others, the higher levels of depersonalization and emotional exhaustion were found in medical students

**Disclosure of Interest:**

None Declared

